# Systematic review of lifestyle interventions to improve weight, physical activity and diet among people with a mental health condition

**DOI:** 10.1186/s13643-022-02067-3

**Published:** 2022-09-09

**Authors:** Tegan Bradley, Elizabeth Campbell, Julia Dray, Kate Bartlem, Paula Wye, Grace Hanly, Lauren Gibson, Caitlin Fehily, Jacqueline Bailey, Olivia Wynne, Kim Colyvas, Jenny Bowman

**Affiliations:** 1 University of Newcastle, University Drive, Callaghan, NSW 2308 Australia; 2 Hunter Medical Research Institute, Lot 1, Kookaburra Cct, New Lambton Heights, NSW 2305 Australia; 3 Hunter New England Population Health, Locked Bag 10, Wallsend, NSW 2287 Australia

**Keywords:** Mental health, Physical health, Chronic disease, Lifestyle interventions, Weight management, Physical activity, Diet

## Abstract

**Background:**

People with a mental health condition experience an elevated risk of chronic disease and greater prevalence of health and behaviours. Lifestyle interventions aim to reduce this risk by modifying health behaviours such as physical activity and diet. Previous reviews exploring the efficacy of such interventions for this group have typically limited inclusion to individuals with severe mental illness (SMI), with a focus of impact on weight. This review assessed the efficacy of lifestyle interventions delivered in community or outpatient settings to people with any mental health condition, on weight, physical activity and diet.

**Methods:**

Eligible studies were randomised or cluster-randomised controlled trials published between January 1999 and February 2019 aiming to improve weight, physical activity or diet, for people with any mental health condition. Two reviewers independently completed study screening, data extraction and assessment of methodological quality. Primary outcome measures were weight, physical activity and diet. Secondary outcome measures were body mass index (BMI), waist circumference, sedentary behaviour and mental health. Where possible, meta-analyses were conducted. Narrative synthesis using vote counting based on direction of effect was used where studies were not amenable to meta-analysis.

**Results:**

Fifty-seven studies were included (49 SMI only), with 46 contributing to meta-analyses. Meta-analyses revealed significant (< 0.05) effect of interventions on mean weight loss (−1.42 kg), achieving 5% weight loss (*OR* 2.48), weight maintenance (−2.05 kg), physical activity (IPAQ MET minutes: 226.82) and daily vegetable serves (0.51), but not on fruit serves (0.01). Significant effects were also seen for secondary outcomes of BMI (−0.48 units) and waist circumference (−0.87cm), but not mental health (depression: *SMD* −0.03; anxiety: *SMD* −0.49; severity of psychological symptoms: *SMD* 0.72). Studies reporting sedentary behaviour were not able to be meta-analysed. Most trials had high risk of bias, quality of evidence for weight and physical activity were moderate, while quality of evidence for diet was low.

**Conclusion:**

Lifestyle interventions delivered to people with a mental health condition made statistically significant improvements to weight, BMI, waist circumference, vegetable serves and physical activity. Further high-quality trials with greater consistency in measurement and reporting of outcomes are needed to better understand the impact of lifestyle interventions on physical activity, diet, sedentary behaviour and mental health and to understand impact on subgroups.

**Systematic review registration:**

PROSPERO CRD42019137197

**Supplementary Information:**

The online version contains supplementary material available at 10.1186/s13643-022-02067-3.

## Introduction

It is estimated that 17% of the population will experience a mental health condition, encapsulating more commonly encountered conditions such as depression and anxiety, as well as less common potentially severe conditions such as schizophrenia and psychotic disorders [1]. People with mental health conditions experience a significant reduction in life expectancy, which has been largely attributed to elevated risk of chronic conditions such as cardiovascular disease and diabetes [2]. These conditions result from health risks such as overweight and obesity, poor nutrition and physical inactivity [3, 4], which have been reported to have a higher prevalence among this group [5–10]. There is a need for evidence of effective interventions to address these health risks for people with a mental health condition [2, 11–13].

Lifestyle interventions are one means of supporting changes to physical health by promoting improvements in weight, physical activity and diet. Such interventions include programmes based on a variety of approaches including provision of health education, and cognitive behavioural therapy (CBT), and may also include practical components such as participation in physical activity. Lifestyle interventions can be delivered in individual or group settings across a range of modalities including face to face, telephone or via web-based methods. Systematic review evidence has shown that among general population samples, lifestyle interventions are an effective means to achieve significant improvements in anthropometric measures including reductions in body weight, body mass index (BMI) and waist circumference [14], as well as improvements to physical activity [15] and dietary outcomes including fruit and vegetable consumption [16]. Lifestyle interventions have also been associated with reduced risk for chronic health conditions such as cardiovascular disease [17], type 2 diabetes and metabolic syndrome [18], as well as all-cause mortality [19].

For the priority population group of people with a mental health condition, systematic review evidence suggests that lifestyle interventions can lead to improvements in anthropometric measures. For example, meta-analyses have indicated that lifestyle interventions for people with severe mental illness (SMI) resulted in significant reductions of up to 2 kg in weight [20–23], 1.2 BMI units [20, 24–26] and 2.5 cm in waist circumference [23, 25] when compared to control conditions. A systematic review using narrative synthesis to assess the impact of lifestyle interventions on physical activity among people with SMI in both controlled and uncontrolled trials found low-quality evidence for both types of trials [27]. Of 16 controlled trials, seven reported significant improvements in physical activity comparative to controls, while 3 of 16 uncontrolled trials reported improvement. However, no systematic reviews could be located that use meta-analyses to synthesise the impact of lifestyle interventions on physical activity among people with a mental health condition. Similarly, no reviews with or without meta-analysis could be located examining the efficacy of lifestyle intervention on improvements in diet among people with a mental health condition. Additionally, previous reviews are limited by typically including only individuals with SMI (for example schizophrenia), who make up a small proportion of the population with mental health conditions [28, 29]. Some reviews include studies across both inpatient and community settings [20, 21, 23, 25], with the possibility of differences in wellness, or participant autonomy with respect to diet or physical activity, potentially impacting results.

To address these gaps in the literature, the aim of the current systematic review was therefore to evaluate the effectiveness of lifestyle interventions delivered in a community or outpatient settings to people with any mental health condition, on weight, physical activity and diet. The review also included secondary outcomes of the impact of interventions on BMI, waist circumference, sedentary behaviour and mental health.

## Methods

### Search methods

A systematic review was reported following the Preferred Reporting Items for Systematic Reviews and Meta-Analyses (PRISMA) statement [30] and prospectively registered with PROSPERO (CRD42019137197). Seven electronic databases were searched from January 1999 to February 2019: PsycINFO, MEDLINE, Excerpta Medica database (EMBASE), Psychology and Behavioural Sciences Collection, Scopus, Cochrane Central Register of Controlled Trials (CENTRAL) and Cumulative Index to Nursing and Allied Health Literature (CINAHL) (Supplementary Table 1. Example search strategy for PsycINFO). This time frame was chosen in order to collect the most recent 20 years of studies prior to being impacted by COVID-19. The search included terms relating to mental health conditions, the three primary outcome measures (weight, physical activity and diet), study designs and intervention types. Authors of included protocol papers or registrations were contacted for any publication of outcomes, and reference lists of included studies and related systematic reviews were hand-searched for potentially eligible studies.

### Study inclusion criteria

#### Study design

Randomised and cluster-randomised controlled trials were eligible for inclusion. Control groups included treatment as usual, brief advice or minimal intervention. Studies with multiple intervention arms were included.

#### Participants

Eligible studies included people with a mental health condition (with the exception of eating-related disorders or neurodegenerative disorders) at the time of recruitment into the trial, indicated via clinician or client reported diagnosis or inferred through current treatment for a mental health condition. Studies in which the mean age of participants was less than 18 years, or in which results were not reported separately for participants over 18 years, were excluded.

#### Interventions

Interventions which promoted and/or supported weight loss/management and/or changes in physical activity and/or diet for individuals, were delivered by any mode (face to face, telephone, digital), delivered to groups or individuals and based on any framework or approach (e.g. education, skills training, CBT, facilitation of behaviours), were included. Interventions which included any pharmacological component other than those for the management of an existing mental health condition (e.g. interventions trialling weight loss drugs) were excluded.

#### Setting

Studies were included if interventions were delivered in community-based settings, such as outpatient services, community managed organisations, shared housing, online or other areas in the general public. Interventions delivered in inpatient settings were excluded.

#### Study outcome measures

Studies were included if they quantitatively assessed at least one of the primary review outcomes: weight, physical activity or diet. Eligible weight measures included weight (kilograms (kg) or pounds (lb)) and assessed via self-report or objective measurement. Studies measuring changes in weight were separated by intervention aim as follows: (1) to reduce the weight of participants in the intervention group (weight loss) or (2) to minimise potential weight gain, for example amongst individuals taking antipsychotic medication known to result in weight gain (weight maintenance). Eligible physical activity measurements included self-report or objective measurement tools (e.g. accelerometer), expressed as any unit of measurement (e.g. number of sessions, number of minutes, number of steps per day/week) and any intensity (e.g. light, moderate, vigorous). Eligible diet measures included an individual’s consumption of any food or beverages over any period of time (mean serves, number of times consumed per day/week, meeting recommendations for food groups) and assessed via tools such as food frequency questionnaires, diaries, records or surveys.

Secondary review outcomes were as follows: body mass index (BMI) [31] based on objective or reported height and weight measures; waist circumference, measured in centimetres or inches based on objective measurement or self-report; sedentary behaviour, as indicated by any measurement of time (e.g. minutes per day, sessions per day, total per week, sitting time); and mental health (any validated measure of mental health).

### Study selection process

Two reviewers independently assessed titles and abstracts of identified studies for eligibility based on predetermined inclusion/exclusion criteria. The full texts of studies that met inclusion criteria were then independently assessed by two reviewers for final inclusion. Consensus was first attempted to resolve any disagreement between the two reviewers regarding study eligibility or, if required, resolved via a third reviewer. Where necessary, corresponding authors were contacted for further details to determine study eligibility.

### Data extraction

Data was extracted for all measures of each review outcome. Two reviewers independently extracted data for each included study using a standardised Word document form, modelled from the Cochrane data extraction template [32]. Disagreements regarding data extraction were resolved through consensus between the two reviewers. Where possible, outcome data were entered into RevMan software to complete meta-analyses [33]. Where outcome data were reported in a format amenable to meta-analysis but necessary data were missing, corresponding authors were contacted for further details.

The following information was extracted:

- Author and year of publication, country, study design, number of trial arms, sample size and percentage female, mean age and mental health condition
- Characteristics of the intervention including the following: health behaviours promoted, intervention and comparison group conditions, duration and method of delivery (e.g. face to face, telephone, multiple)
- All data pertaining to primary and secondary outcomes including data measurement tools, effect summary statistics and measures of outcome variability

### Data analysis and synthesis

#### Meta-analysis

Studies were first examined for outcomes reported in a manner suitable for inclusion in meta-analyses. Random effect meta-analyses were conducted where at least three studies measured an outcome using the same or comparable measures or format. Intervention effect sizes and 95% confidence intervals were calculated. If a study had multiple measures for the same review outcome, one data source was selected according to the following hierarchy: (1) a score for a full tool (e.g. global score) was used rather than subscale; (2) where two tools measured the same outcome, the tool with greater validity was used; (3) where outcome measures were identified as primary or secondary by the study authors, the primary outcome was chosen; and (4) where no outcome was explicitly identified as primary, the measure consistently described first in the measures and/or results tables was used.

Random effects meta-analysis models were used with the RevMan 5 software [33] to calculate study effect size for mean difference, odds ratios and standardised mean differences. Where a study had multiple trial arms, the most comprehensive intervention (e.g. with greatest frequency of contact) was compared to the control condition. Where a study provided data for more than one follow-up point, the time point closest to the end of the intervention was used. When studies did not provide mean differences and their standard deviations for intervention and control groups, a range of approaches were used to derive the required information from what study authors provided [34]. One set of approaches involved imputing the size of the correlation coefficient between pre and post scores for the calculation of the standard deviation of the mean difference. For meta-analyses in which the conservative estimate of 0 for the correlation coefficient was not used (weight, waist circumference, BMI), a sensitivity analysis was carried out to determine the effect of the choice of correlation coefficient on the overall meta-analysis study effect size. See Supplementary Table 2 for details on approaches employed during meta-analyses and the sensitivity analyses.

Due to an insufficient number of studies [35] or insufficient detail of intervention approach, subgroup analyses based on mental health condition, delivery method and health behaviours targeted by the intervention specified a priori were not undertaken (PROSPERO ID: CRD42019137197).

#### Narrative synthesis

A narrative synthesis using vote counting for direction of effect was used to summarise evidence of effect and supplement the meta-analytic findings [34] where studies are as follows:

1. Presented an outcome in a format that was not conducive to meta-analysis (e.g. in a manner not comparable to other studies or insufficient studies measuring in the same format to form a meta-analysis)
2. Insufficient data was able to be received from authors for inclusion in a meta-analyses.

If a study presented data in multiple formats corresponding with more than one ‘measure’ under the one review outcome (e.g. weight change in kg, proportion of sample achieving 5% weight loss), data from the study could be included in a meta-analysis for one measure and in narrative synthesis for the other measure.

### Assessment of risk of bias and quality of cumulative evidence

Risk of bias for each included study was assessed independently by two reviewers against the revised Cochrane risk-of-bias tool [36]. Study characteristics including selection bias (sequence generation and allocation concealment), performance bias (blinding of participants and personnel), detection bias (blinding of outcome assessment), attrition bias (no incomplete outcome data), reporting bias (no selective reporting) and other potential sources of bias were assessed [34]. Risk of bias for cluster-randomised trials was assessed against additional criteria, including recruitment to cluster, baseline imbalance, loss of clusters and incorrect analysis [34]. Grading of Recommendations Assessment, Development and Evaluation (GRADE) approach was used to assess confidence in cumulative evidence for each primary review outcome [37–39].

## Results

### Study selection

**Fig. 1:**
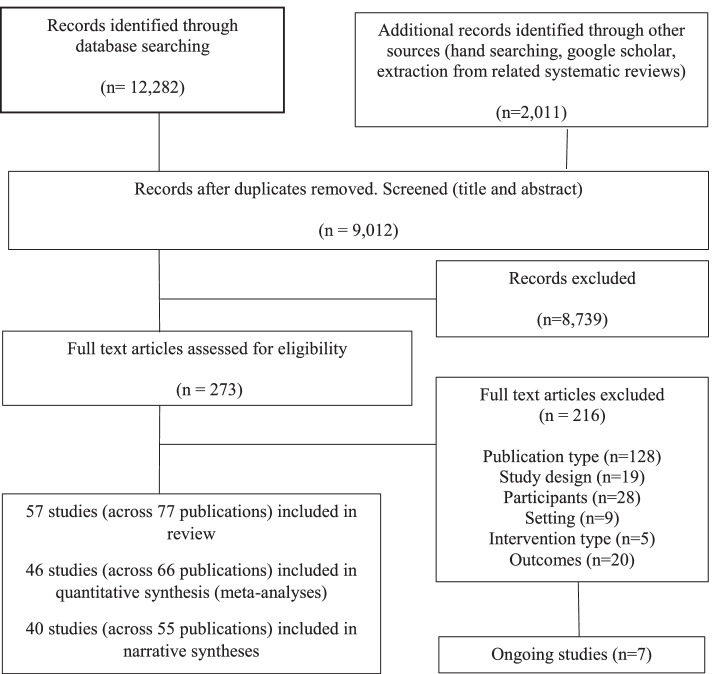
Selection of studies. NB, studies may contribute to both quantitative and narrative synthesis for different variables

A total of 14,293 studies were collected via search strategies. Following the removal of duplicates, the search identified 9012 records, with 8739 studies excluded at title and abstract screening and a further 216 excluded during full-text review (Fig. 1). A total of 57 studies (52 randomised controlled trials and 5 cluster-randomised controlled trials) were included in the review.

### Characteristics of included studies

Included studies were published between January 2003 and February 2019 and conducted across 13 countries, with the largest number conducted in USA (*n* = 23), followed by UK (*n* = 11) (Table 1). Studies included participants with a range of mental health conditions, with the most commonly reported conditions being schizophrenia and schizoaffective disorder and depression. Due to variability in the inclusion criteria of diagnoses considered be SMI, a broad classification was used guided by classifications used by previous systematic reviews [20, 25]. In addition, any study which identified that it considered its participants to have an SMI, or that included only participants who were taking antipsychotic medication, were classified as SMI studies. Of the 57 included studies, 49 included only participants considered to have an SMI, 6 included only participants considered to not have an SMI, and 2 included participants both with and without an SMI in their sample. Intervention duration ranged from 1 week to 12 months, with the most common duration (*n* = 18) being 12 weeks and 48 having intervention of 12 weeks or longer. The majority of interventions were delivered in face-to-face format (*n* = 44), followed by mixed mode of face to face and phone (*n* = 9), web/computer (*n* = 3) and phone only (*n* = 1). A total of 37 studies had interventions that promoted healthy weight, 34 promoted physical activity, 14 promoted diet and 23 promoted more than one of these. Sixteen interventions also promoted additional health issues not captured in this review, including smoking (*n* = 7) and alcohol consumption (*n* = 4). A total of 36 studies reported changes in weight, with 31 measured as weight loss and 5 measured as weight maintenance (minimisation of weight gain while taking antipsychotic medication). Physical activity was measured in 35 studies and diet in 14. BMI was measured in 34 studies and waist circumference in 23. Sedentary behaviour was measured in three studies and mental health outcomes in 24.

**Table 1:** Characteristics of included studies

Author, year, country	Design, no. of trial arms	Participants: no. analysed, mean age, % female mental health diagnoses, SMI inclusion^a^	Health behaviours or variables promoted as a part of intervention	Intervention description (length, delivery mode) Control/comparison group	Primary review outcomes assessed Weight, physical activity (PA), diet	Secondary review outcomes assessed Body mass index (BMI)/waist circumference (WC)/mental health (MH)/sedentary behaviour
Abrantes, 2017, USA [40, 41]	RCT, 2	56, 39, 64%, obsessive-compulsive disorder (SMI only)	Physical activity	Intervention: weekly education and exercise sessions plus US $5 reimbursement for each session attended. Participants were also instructed to exercise on their own between 2 and 4 days during the week (12 weeks, F2F) Control: weekly hourlong psychoeducation sessions	PA: IPAQ	MH: Beck Anxiety Inventory, Beck Depression Inventory, Yale-Brown Obsessive-Compulsive Scale
Alvarez-Jimenez, 2006, Spain [42]	RCT, 2	61, 27, 25%, first episode psychosis (SMI only)	Weight, physical activity, diet	Intervention: 10 to 14 sessions to develop strategies to enhance control over factors associated with antipsychotic-induced weight gain such as energy intake and activity (3 months, F2F) Control: provide patients with the same physical care that is offered in a comprehensive early psychosis programme	Weight: maintenance (kg)	BMI
Attux, 2013, Brazil [43]	RCT, 2	126, intervention, 36 control, 39, 40%, schizophrenia, other psychosis (SMI only)	Physical activity, diet, mental health	Intervention: lifestyle wellness programme consisting of weekly sessions discussing various health measures and behaviours (12 weeks. F2F) Control: TAU	Weight: maintenance (kg) PA: IPAQ	BMI WC: cm
Baker 2018, Australia [44–46]	RCT, 2	139, 41.6, 41%, schizophrenia spectrum disorder, bipolar disorder, nonorganic psychotic syndrome (SMI only)	Physical activity, diet Smoking, alcohol	Intervention: smoking cessation support including NRT, plus 16 sessions of CBT focussing on CVD risk factors considered most problematic by the participant (30 weeks, F2F and phone) Control: smoking cessation support including NRT only	PA: IPAQ Diet: study-specific question	WC: cm MH: Beck Depression Inventory; Brief Psychiatric Rating Scale; Global Assessment of Functioning Sedentary behaviour: IPAQ
Bartels, 2013, USA [47]	RCT, 2	104, 44, 62%, schizophrenia, schizoaffective disorder, bipolar disorder, major depression, other (SMI only)	Weight, physical activity, diet	Intervention: in SHAPE programme. Fitness club membership and health promotion coach. (12 months, F2F) Control: fitness club membership to the same local fitness clubs (YMCAs)	Weight: loss (lb) PA: IPAQ	BMI
Bartels, 2015, USA [48]	RCT, 2	210, 44, 51%, schizophrenia, schizoaffective disorder, bipolar disorder, major depression (SMI only)	Physical activity, diet	Intervention: in SHAPE programme. Fitness club membership and health promotion coach (12 months, F2F) Control: fitness club membership to the same local fitness clubs (YMCAs)	Weight: loss (lb) PA: IPAQ Diet: Brief Block Food Frequency Questionnaire	BMI WC: in
Battaglia, 2015, Italy [49]	RCT, 2	18, 35, 0%, schizophrenia, schizoaffective disorder (SMI only)	Physical activity	Intervention: two soccer sessions per week. Every training session was approximately 100–120 min (12 weeks, F2F) Control: TAU	Weight: maintenance (kg)	BMI
Beebe, 2011, USA [50]	RCT, 2	79, 47, 47%, schizophrenia, schizoaffective disorder (SMI only)	Physical activity	Intervention: WALC-S, 4 group discussions of the basics of walking for exercise and information on walking safely. Walking groups three times a week (16 weeks, F2F) Control: 4 group sessions focused on other health behaviours. Walking groups three times a week	PA: study specific	
Bersani, 2017, Italy [51]	RCT, 2	32, 52, 50%, schizophrenia, bipolar disorder, major depressive disorder (SMI only)	Physical activity, diet Sleep, smoking, alcohol,	Intervention: five sessions of group psychoeducation on developing and maintaining a healthy lifestyle, booklet of information and promotion of self-monitoring (5 weeks, F2F) Control: five sessions to discuss clinical outcomes, watch and discuss a movie or receive education information on pharmacological therapy	PA: IPAQ Diet: Questionnaire of Mediterranean Diet Adherence	MH: Brief Psychiatric Rating Scale
Bonfioli, 2018, Italy [52]	RCT, 2	302, intervention 45, control 48, 47%, schizophrenia and other functional psychoses, affective psychoses (SMI only)	Physical activity, diet	Intervention: seven health education group sessions, plus weekly group walking sessions under the guidance of an expert trainer (6 months, F2F) Control: TAU	PA: study specific Diet: study specific	
Brown, 2006, UK [53]	RCT, 2	17, intervention 45, control 42, 86%, psychosis, major affective illness, severe personality disorder (SMI only)	Weight, physical activity, diet Smoking, other substance use	Intervention: six individual health promotion sessions, following the Lilly “Meaningful Day” manual, tailored to the needs of the individual (6 weeks, F2F) Control: TAU	Weight: loss (kg) PA: Godin leisure-time questionnaire	BMI MH: Hamilton Anxiety and Depression Scale
Brown, 2009, UK [54]	RCT, 2	26, intervention 50, control 47, 69%, serious mental illness (SMI only)	Weight, physical activity, diet Smoking, other substance use	Intervention: five semi-structured sessions of supervised health promotion interventions from the Lilly “Meaningful Day” package (10 weeks, F2F) Control: TAU	Weight: loss (kg) PA: Godin leisure-time questionnaire Diet: Dietary Instrument for Nutrition Education	MH: Hamilton Anxiety and Depression
Brown, 2011, USA [55]	RCT, 2	89, 44, 61%, serious mental illness (SMI only)	Weight, physical activity, diet	Intervention: RENEW programme. Weekly sessions in which participants learn about nutrition, participate in physical activity, set individualised goals and eat a meal together (3 months, F2F + phone) Control: TAU	Weight: loss (lbs)	
Chalder, 2012, UK [56, 57]	RCT, 2	222, intervention 41, control 39, 66%, depression (non-SMI)	Physical activity	Intervention: maximum of 13 sessions between the patient and the physical activity facilitator, who helped patients set personal targets about incorporating physical activity into their lifestyle with the gradual building up of physical activity as a regular behaviour (8 months, F2F + phone) Control: TAU	PA: study-specific question	MH: Beck Depression Inventory
Chao 2011, USA [58]	RCT, 3	53, 46.8, 33%, major depressive disorder, bipolar disorder, schizophrenia spectrum disorder, posttraumatic stress disorder, anxiety disorder, NOS (SMI only)	Physical activity	Intervention: pedometer use with self-monitoring through daily logbook recordings (2 weeks, F2F) Control: TAU Comparison 2: pedometer use with seal to avoid self-monitoring of daily step count	Weight: loss (kg) PA: IPAQ	MH: Center for Epidemiologic Studies Depression Scale
Daley, 2008, UK [59]	RCT, 2	31, np, 100%, post-natal depression (non-SMI)	Physical activity	Intervention: two consultations and two phone calls, aimed to equip individuals with the skills, knowledge and confidence needed to participate in regular exercise. Checklist exercise diary and pedometer also provided (12 weeks, F2F and phone) Control: TAU	PA: Godin leisure-time questionnaire	MH: Edinburgh Postnatal Depression Scale
Daley, 2015, UK [60, 61]	RCT, 2	85, intervention 32, control 29, 100%, post-natal depression (non-SMI)	Physical activity	Intervention: two personalised consultations centred on equipping women with the skills, knowledge and confidence needed to participate in regular exercise. Information leaflets, telephone support calls and a pedometer were also provided (6 months, F2F + phone) Control: sent the study “looking after yourself” leaflet at baseline and exercise was not further encouraged beyond receipt of this single leaflet	Weight: loss (kg) PA: IPAQ	BMI MH: Edinburgh Postnatal Depression Scale
Daumit, 2013, USA [62]	RCT, 2	288, 45, 50%, schizophrenia, schizoaffective disorder, bipolar disorder, major depression, other, (SMI only)	Weight, physical activity, diet	Intervention: three contact types: group weight-management sessions, individual weight-management sessions and group exercise (6 months, F2F) Control: standard nutrition and physical-activity information. Health classes offered quarterly, with content unrelated to weight	Weight: loss (kg)	BMI WC: cm
Druss, 2010, USA [63]	RCT, 2	65, 48, 70%, bipolar disorder, schizophrenia, major depression, posttraumatic stress disorder (SMI only)	Physical activity, diet Medication management, chronic disease management	Intervention: monthly group sessions led by mental health peer specialists (6 months, F2F) Control: TAU	PA: Behavioural Risk Factor Surveillance System	
Druss, 2018, USA [64]	RCT, 2	400, 50, 64%, schizophrenia, schizoaffective disorder, bipolar disorder, depression, obsessive-compulsive disorder, posttraumatic stress disorder (SMI only)	Physical activity, diet	Intervention: 6 group and one-on-one peer coaching sessions. Structured manual and worksheets to support development of key competencies and skills (np, F2F) Control: TAU	Diet: Block Fat-Sugar-Fruit-Vegetable Screener	
Erickson, 2016, USA [65]	RCT, 2	108, 50, 11%, schizophrenia, schizoaffective disorder, bipolar disorder, other (SMI only)	Physical activity, diet	Intervention: 8 weekly education classes, followed by monthly booster classes for the remainder of the year. Small rewards (e.g. US $10 gift certificates) were provided for achieving weight loss and exercise goals. Group exercise activities led by instructors offered but optional (12 months, F2F) Control: participants were encouraged to exercise and eat healthy and were given publicly available, printed self-help materials regarding weight loss, exercise and nutrition	Weight: 5% loss	
Erickson, 2017, USA [66]	RCT, 2	104, 51, 19%, schizophrenia, schizoaffective disorder, bipolar disorder, other (SMI only)	Weight, physical activity, diet	Intervention: 8 weekly education classes, followed by monthly booster classes for the remainder of the year, as well as individual nutrition counselling (12 months, F2F) Control: self-help educational handouts on health issues	Weight: loss (kg) PA: study specific	WC (cm)
Evans, 2005, Australia [67]	RCT, 2	34, 34, 57%, schizophrenia, schizoaffective disorder, schizophreniform psychosis, bipolar disorder, depression (SMI only)	Physical activity, diet	Intervention: six individual nutrition education sessions plus passive nutritional education from the booklet Food for the Mind (3 months, F2F) Control: Food for the Mind booklet only	Weight: loss (kg)	BMI WC: cm
Forsberg, 2008, Sweden [68]	CRCT, 2	35, intervention 40, control 43, 39%, schizophrenia, bipolar disorder, other psychotic disorder, other psychiatric diagnosis (SMI only)	Physical activity, diet	Intervention: study circles (5–12 residents) twice a week for one session on the diet and the other on physical activity (12 months, F2F) Control: aesthetic study circle as attention control focusing on non-health-related skills	Weight: loss (kg) PA: pedometer	BMI WC: cm
Forsyth, 2015, Australia [69]	RCT, 2	94, range: 18–84, 28%, depression, anxiety (non-SMI)	Physical activity, diet	Intervention: based on Chronic Disease Management Plan Six visits to a dietician/exercise physiology (12 weeks, F2F) Control: participants received phone calls from the DEPs at similar intervals to the intervention consultations, but no advise	Weight: loss (kg) PA: Active Australia Survey Diet: Diet History Questionnaire	BMI MH: Depression Anxiety and Stress Scale
Gaughran, 2017, UK [70, 71]	CRCT, 2	325, 44, 42% psychotic disorder (SMI only)	Physical activity, diet Smoking, alcohol, other substance use	Intervention: patient-tailored IMPACT therapy, supported by a manual, a reference book and a service user handbook. Participants had the option to receive 3-monthly newsletters throughout the trial period (12 months, F2F) Control: all care coordinators in participating CMHTs offered best practice treatment as usual training on physical health awareness, including the provision of health promotion leaflets on healthy dietary routines and physical exercise, together with information on general and community support for a healthy lifestyle	PA: IPAQ	BMI WC: cm MH: Positive and Negative Syndrome Scale, Global Assessment of Functioning, Montgomery-Asberg Depression Rating Scale
Gillhoff, 2010, Switzerland [72]	RCT, 2	50, 48, 46%, bipolar disorder (SMI only)	Weight, physical activity, diet	Intervention: quality of life for persons with bipolar disorder programme. Seven lifestyle sessions, 4 nutrition sessions and weekly instructions pertaining to physical activity (5 months, F2F) Control: TAU	Weight: loss (kg)	BMI WC: cm
Goldberg, 2013, USA [73]	RCT, 2	71, 52, 19%, schizophrenia or schizoaffective disorder, major depression, bipolar disorder, posttraumatic stress or anxiety disorder (SMI only)	Weight, physical activity, diet	Intervention: *MOVE!* programme. Psychoeducation including individual and group face-to-face counselling and telephone care individual sessions (6 months, F2F) Control: brochures and handouts about diet and exercise	Weight: loss (lb) PA: study specific Diet: study-specific question	WC: np
Goracci, 2016, Italy [74]	RCT, 2	160, 49, 80%, bipolar disorder, recurrent unipolar depression (mixed)	Weight, physical activity, diet, mental health Sleep	Intervention: weekly sessions based on a comprehensive, standardized and integrated manualized programme. All information was adapted to both unipolar and bipolar patients and to Italian culture and habits (3 months, F2F) Control: TAU	Weight: loss (kg)	BMI WC: cm MH: Patient Health Questionnaire-9, Clinical Global Impression — Severity of Illness
Green, 2014, USA [75]	RCT, 2	36, 49, 81%, np (taking antipsychotic medication) (SMI only)	Weight, physical activity, diet	Intervention: PREMIER, with adaptations. Weekly group sessions, including a half hour walk. Food and exercise diaries encouraged (12 weeks, F2F) Control: TAU	Weight: loss (lbs)	
Green, 2015, USA [76–79]	RCT, 2	200, 47, 72%, schizophrenia spectrum disorder, bipolar disorder or affective psychosis, posttraumatic stress disorder (SMI only)	Weight, physical activity, diet Sleep, blood pressure	Intervention: STRIDE. Weekly group meetings with 20 min of physical activity, supplemented by monthly individual telephone sessions. Participants were taught to keep records of food intake and received a workbook and a resistance band for strength training (12 months, F2F + phone) Control: TAU	Weight: loss (kg)	BMI
Gyllensten, 2017, Sweden [80]	CRCT, 2	73, 38, 41%, schizophrenia, Neuropsychiatric disorder, psychosis other, bipolar disorder, other (SMI only)	Physical activity	Intervention: exergames controlled by body movements integrated into supported housing (10 months, NA) Control: ordinary TV games in sitting position controlled by hand control	PA: physical activity habits	
Holt, 2019, UK [81, 82]	RCT, 2	340, 40, 49%, schizophrenia, schizoaffective disorder, first-episode psychosis (SMI only)	Weight, physical activity, diet	Intervention: STEPWISE intervention. Groups of participants attended a foundation course of four weekly sessions, followed by 1:1 support contact, mostly by telephone, approximately every 2 weeks for the remainder of the intervention period (12 months, F2F + phone) Control: printed advice on lifestyle and the risks associated with weight gain	Weight: loss (kg), % maintained or lost weight PA: accelerometer	BMI WC: cm MH: Brief Psychiatric Rating Scale, Patient Health Questionnaire-9
Iglesias-Garcia, 2010, Spain [83]	RCT, 2	14, 40, 21%, schizophrenia (SMI only)	Physical activity, diet	Intervention: structured educative programme provided information and counselling on three domains: nutrition, exercise and healthy habits and self-esteem. Weekly sessions included structured information given to patients and group discussion lead by the nurse about any aspect of the programme (3 months, F2F) Control: attended to the clinic once a week to assess the anthropometric parameters	Weight: loss (kg)	BMI WC: cm
Jacka, 2017, Australia [84, 85]	RCT, 2	56, 40, 72%, severe depression (SMI only)	Diet, mental health	Intervention: 7 dietary support sessions, with a focus on increasing diet quality (12 weeks, F2F) Control: “befriending” as a form of attention control	PA: IPAQ Diet: ModiMedDiet	BMI MH: Montgomery-Asberg Depression Rating Scale, Hospital Anxiety and Depression Scale, Clinical Global Impression — Improvement Scale
Jean-Baptiste, 2007, USA [86]	RCT, 2	14, 47, 50%, schizophrenia, schizoaffective disorder (SMI only)	Weight, physical activity, diet	Intervention: weekly sessions using principles based on the LEARN programme. Exercise was encouraged and pedometers provided. Participants were provided with a specific listing of foods they could purchase and that they would be reimbursed for, up to US $25 a week (16 weeks, F2F) Control: TAU	Weight: loss (lb)	
Khazaal, 2007, Switzerland [87]	RCT, 2	48, 41, 54%, schizophrenia, schizoaffective disorder, bipolar disorder, schizotypal disorder, other (depression, personality disorders) (SMI only)	Weight, diet	Intervention: weekly sessions. The apple-pie group was conceived as a handbook for a CBT treatment for severe psychiatric patients and adopts techniques such as Socratic questioning suited for patients with psychotic disorders (12 weeks, F2F) Control: an informative 2-h group session. At the end of the session, patients were given nutritional recommendations in the form of a written summary and were encouraged to refer frequently to these guidelines and maintain their effort to lose weight	Weight: loss (kg)	BMI
Kilbourne, 2017, USA [88, 89]	RCT, 2	245, 55, 15%, schizophrenia, bipolar disorder, major depressive disorder, other SMI diagnosis (SMI only)	Physical activity, diet CVD risk behaviours	Intervention: Life Goals Collaborative Care included a self-management component, with 5 weekly group sessions, a care management component consisting of 6 monthly contacts and provider support in which the health specialist disseminates a care plan that includes patients’ health status and behaviour goals to their primary care and mental health providers after the last care management contact (12 months, F2F + phone) Control: TAU	PA: IPAQ	BMI WC: in MH: Patient Health Questionnaire-9, Generalized Anxiety Disorder 7-item, PTSD CheckList — Civilian Version, Internal State Scale (ISS), Behavior Symptom Identification Scale
Kwon, 2006, Korea [90]	RCT, 2	43, intervention 32, control 30, 52%, schizophrenia, schizoaffective disorder (SMI only)	Weight, physical activity, diet	Intervention: 8 sessions including education about keeping food and exercise diaries, eating behaviour improvement and lifestyle modification for weight control (12 weeks, F2F) Control: Food & exercise diaries only	Weight: loss (kg)	BMI
Lee, 2014, USA [91]	RCT, 2	16, 44, 46%, Schizophrenia spectrum disorders, bipolar disorder, major depression (SMI only)	Physical activity, BMI, waist circumference Blood pressure	Intervention: weekly phone calls, involving behavioural counselling for physical activity that included goal setting, self-monitoring, feedback on goal achievement and social support. Pedometer supplied (8 weeks, phone) Control: written information regarding physical activity (e.g. exercise suggestions)	PA: IPAQ	BMI WC: in
Littrell, 2003, USA [92]	RCT, 2	70, 34, 39%, schizophrenia, schizoaffective disorder (SMI only)	Weight, physical activity, diet	Intervention: weekly psychoeducation classes using “Solutions for Wellness” modules (16 weeks, F2F) Control: TAU	Weight: maintenance (lb)	BMI
Lovell, 2014, UK [93]	RCT, 2	93, 26, 40%, schizophrenia, schizoaffective disorder, schizophrenia spectrum disorder, NOS (SMI only)	Weight, physical activity, diet	Intervention: 8 individual psychoeducation sessions, including optional group activities, plus a booklet and website to provide additional support (12 months, F2F) Control: TAU	Weight: loss (kg) PA: IPAQ Diet: food frequency questionnaire	BMI WC: cm MH: Calgary Depression Scale
Marzolini, 2009, Canada [94]	RCT, 2	13, 45, 38%, schizophrenia, schizoaffective disorder (SMI only)	Physical activity, mental health	Intervention: twice weekly supervised group exercise sessions (12 weeks, F2F) Control: TAU	Weight: loss (kg)	BMI WC: cm MH: Mental Health Inventory
Masa-Font, 2015, Spain [95, 96]	RCT, 2	332, 47, 45% schizophrenia, schizoaffective disorder, bipolar disorder (SMI-only)	Physical activity, diet	Intervention: 8 group sessions focusing on physical activity, with the aim for participants to reach 10,000 steps per day, plus 16 group sessions providing basic knowledge about healthy dietary habits, with a focus on a Mediterranean diet for cardiovascular protection. Included food diary (6 months, F2F) Control: TAU	PA: IPAQ Diet: PREvención con DIetaMEDiterránea” (PREDIMED)	BMI WC: cm MH: Clinical Global Impressions Scale
Mauri, 2008, Italy [97]	RCT, 2	33, 39, 58%, bipolar I disorder, bipolar II disorder, schizoaffective disorders, psychotic depression SMI only	Physical activity, diet	Intervention: psychoeducation programme consisting of consecutive intensive weekly meetings with the goal of obtaining a weight loss of 2 kg/month. The programme was adapted to psychiatric patients by simplifying some steps (12 weeks, F2F) Control: TAU	Weight: loss (kg)	BMI MH: Global Assessment Scale of Functioning
McCreadie, 2005, UK [98]	CRCT, 3	91, 45, 29%, schizophrenia (SMI only)	Diet	Intervention: free fruit and vegetables provided for a period of 6 months and associated instructions. Such instruction included meal planning and the purchase, storage and preparation of food, with particular reference to fruit and vegetables (6 months, F2F) Control: TAU Comparison 2: receive free fruit and vegetables for a period of 6 months (no instructions)	Diet: Scottish Health Survey Questionnaire	MH: Positive and Negative Syndrome Scale
McKibbin, 2006, USA [99, 100]	RCT, 2	57, intervention 53, control 55, 35%, schizophrenia, schizoaffective disorder (SMI only)	Physical activity, diet Blood sugar	Intervention: Diabetes Awareness and Rehabilitation Training (DART) comprised a 24-week intervention with three modules. Participants met in groups with 6 to 8 of their peers and one diabetes-trained mental health professional (6 months, F2F) Control: 3 brochures from the American Diabetes Association relevant to diabetes management	Weight: loss (lb) PA: accelerometer, Yale Physical Activity Scale	BMI WC: in
Muralidharan, 2018, USA [101, 102]	RCT, 3	207, intervention 56, control 54, comparison 54, 4%, schizophrenia, schizoaffective disorder, bipolar disorder, major depressive disorder with psychosis, posttraumatic stress disorder (SMI only)	Weight, physical activity, diet	Intervention: two online modules per week (6 months, web) Control: educational handout on the benefits of weight loss Comparison 2: *Move SMI*. Same content as intervention but in F2F format	Weight: loss (kg), 5% loss PA: IPAQ	BMI
Osborn, 2018, UK [103, 104]	CRCT, 2	289, 51, 53%, schizophrenia, schizoaffective disorder, bipolar affective disorder, other psychoses (SMI only)	Weight, physical activity, diet Smoking, alcohol, other CVD risk measures	Intervention: weekly or fortnightly appointments with a nurse or health-care assistant to develop and discuss goals to lower CVD risk (6 months, F2F) Control: TAU + British Heart Foundation leaflets	PA: IPAQ	BMI WC: cm
Pentecost, 2015, UK [105, 106]	RCT, 2	44, 44, 48%, depression (non-SMI)	BMI, mental health, sedentary behaviour Sleep, blood pressure	Intervention: behavioural activation programme consisting of up to 12 support sessions, plus physical activity promotion (4 months, F2F and/or phone) Control: behavioural activation programme only	PA: accelerometer	MH: Clinical Interview Schedule-Revised, Patient Health Questionnaire-9 Sedentary behaviour: Accelerometer
Petzold, 2018, Germany [107]	RCT, 2	83, 49, 72%, mental and behavioural disorders due to psychoactive substance abuse, schizophrenia, schizotypal and delusional disorders, mood disorders, neurotic stress-related and somatoform disorders (mixed)	Physical activity, diet, mental health	Intervention: MoVo-LISA, a psychological group intervention to increase physical activity. Two group sessions and one single session to discuss benefits and barriers to physical activity, individual goals and plans (1 week, F2F) Control: a healthy diet intervention which used behavior change strategies exactly MoVo-LISA but targeted a healthy diet instead of physical activity.	PA: IPAQ, pedometer Diet: dietary pattern index	
Ratliff, 2012, USA [108]	RCT, 3	26, intervention 49, control 47, comparison 50, 43%, schizophrenia, schizoaffective disorder, NOS (SMI only)	Weight, physical activity, diet	Intervention: SIMPLE programme, specifically designed to be used with SMI populations. Individuals in the contingency management attendance group received monetary reward for each of the 8 weight loss groups they attended (8 weeks, F2F) Control: instructed to continue with their habitual patterns of eating and activity Comparison: SIMPLE programme, received monetary reward for weight loss	Weight: loss (kg) PA: Godin leisure-time questionnaire	BMI WC: cm
Skinar, 2005, USA [109]	RCT, 2	20, intervention, 40 control, 36, 66%, mood or psychotic disorder (SMI only)	Weight, physical activity, diet Stress	Intervention: exercise sessions (4 p/week) + weekly health seminars (12 weeks, F2F) Control: asked to journal their physical activity	Weight: loss (kg)	BMI MH: Symptom Checklist — 90
Speyer, 2016, Denmark [110–112]	RCT, 3	428, 39, 56%, schizophrenia, schizoaffective disorder, persistent delusional disorder (SMI-only)	Physical activity, diet Smoking	Intervention: CHANGE lifestyle coaching. Weekly home visits with systematic exploration of possibilities for physical activity in daily life (12 months, F2F) Control: TAU Comparison 2: CARE. Care coordinator facilitated contact to primary care in order to ensure that the patients received optimal treatment of physical health problems	Weight: loss (kg) PA: Physical Activity Scale Diet: dietary quality score	BMI WC: cm MH: Scale for the Assessment of Positive Symptoms, Scale for the Assessment of Negative Symptoms Sedentary behaviour: Physical Activity Scale
Strom, 2013, Sweden [113]	RCT, 2	48, 49, 83%, depression (non-SMI)	Physical activity	Intervention: weekly modules in a guided self-help programme administered through an Internet-based system. Pedometers provided (9 weeks, web) Control: wait list	PA: IPAQ	MH: Montgomery-Asberg Depression Rating Scale, Beck Depression Inventory: Second Version, Beck Anxiety Inventory
Usher, 2013, Australia [114, 115]	RCT, 2	101, np, 47%, schizophrenia, depression, bipolar disorder, anxiety, (SMI only)	Weight, physical activity, diet, BMI, waist circumference Medication compliance	Intervention: weekly education sessions and discussion on healthy lifestyle topics and participants’ progress with the implementation of healthy lifestyle components into their everyday life (12 weeks, F2F) Control: education booklet	Weight: loss (kg)	BMI WC: cm
Weber, 2006, USA [116]	RCT, 2	15, np, 71%, schizophrenia, schizoaffective disorder (SMI only)	Weight, physical activity, diet	Intervention: weekly group sessions based on cognitive/behavioural strategies to promote risk reduction that was demonstrated successful in the Diabetes Prevention Project (DPP). Each person kept a food and activity diary which was turned in at the beginning of each session (16 weeks, F2F) Control: TAU	Weight: loss (lb)	BMI

*Abbreviations*: severe mental illness (*SMI*), randomised controlled trial (*RCT*), cluster-randomised controlled trial (*CRCT*), not otherwise specified (*NOS*), cardiovascular disease (*CVD*), treatment as usual (*TAU*), International Physical Activity Questionnaire (*IPAQ*), Face to Face (*F2F*), not reported (*np*), centimetres (*cm*), inches (*in*), kilograms (*kg*), pounds (*lb*). ^a^“SMI only” indicates that all participants within this study have a severe mental illness. “Mixed” indicates study samples included both participants with and without an SMI. “Non-SMI” indicates that the study only includes participants without an SMI

### Primary outcomes

Table 2 provides a summary of findings for meta-analyses and narrative syntheses. Forest plots for each meta-analysis can be found in supplementary Figs. 1–11. A detailed description of findings for narrative syntheses can be found in Supplementary Table 3.

**Table 2:** Summary of findings from meta-analyses and narrative syntheses

*Outcomes*	Meta-analysis		Narrative synthesis	
	*No. of participants (trials)*	*Effect size*^*a*^ *[95% CI]*	*No of trials*	*Direction of effect — number of studies*
Primary review outcomes				
**Weight**				
Weight loss (kg)	2386 (26)	**−1.42 [−2.21 to −0.63]**	9	Intervention 8 Neither 1
Weight loss (5% bodyweight)	809 (5)	***OR*** **2.48 [1.74 to 3.55]**	-	-
Weight maintenance (kg)	376 (5)	**−2.05 [−3.40 to −0.70]**	1	Intervention 1
**Physical activity**				
International Physical Activity Questionnaire (IPAQ) – total MET minutes	1498 (11)	**226.82 [22.03 to 431.61]**	-	-
Other physical activity measures^b^	-	-	25	Intervention 18 Control 5 Neither 2
**Diet**				
Fruit (daily serves)	337 (3)	0.01 [−0.29 to 0.31]	-	-
Vegetables (daily serves)	367 (3)	**0.51 [0.11 to 0.92]**	-	-
Other diet measures^c^	-	-	13	Intervention 11 Control 1 Neither 1
Secondary review outcomes				
**BMI**				
BMI loss (units)	3919 (33)	−**0.48 [**−**0.70 to** −**0.26]**	1	Intervention 1
**Waist circumference**				
Waist circumference (cms)	3099 (19)	−**0.87 [**−**1.60 to** −**0.14]**	4	Intervention 2 Neither 2
**Sedentary behaviour**				
Sedentary behaviour	-	-	3	Intervention 2 Neither 1
**Mental health**				
Depression	1691 (12)	SMD −0.03 [−0.14 to 0.08]	2	Intervention 2
Anxiety	459 (5)	SMD −0.49 [−1.15 to 0.16]	2	Intervention 2
Severity of psychological symptoms	670 (4)	SMD 0.72 [−0.05 to 1.50]		
Other mental health measures^d^	-	-	14	Intervention 9 Control 5

^a^Unless otherwise noted, the effect sizes are mean differences. ^b^Examples of other measures of physical activity included accelerometers/pedometers and validated self-report measures (e.g. Godin leisure-time questionnaire). See Supplementary Table 3 for full list. ^c^Examples of other measures of diet included meeting the World Health Organization guidelines for fruit and vegetable consumption and comprehensive diet questionnaires such as the food frequency questionnaire. See Supplementary Table 3 for full list. ^d^Examples of other measures of mental health include Global Assessment of Functioning and Edinburgh Postnatal Depression. See Supplementary Table 3 for full list

#### Weight

##### Weight loss

Of the 31 studies that measured weight loss, 26 were included in meta-analysis (23 SMI only; 2 non-SMI; 1 mixed). Meta-analysis of data indicated a positive effect of interventions to reduce weight, with an average weight loss relative to control condition across studies of −1.42 kg (95% *CI* [−2.21 to −0.63]). Five studies measured proportions of participants who achieved at least 5% weight loss (all SMI only), with an odds ratio of 2.48 (1.74 to 3.55) when compared to control conditions. Of the nine studies that measured weight loss included within a narrative synthesis, 8 indicated a direction in favour of the intervention and 1 in neither direction.

##### Weight maintenance

Of the 5 studies that measured weight maintenance, 4 were included in the meta-analysis (all SMI only). Across these studies, intervention participants’ weight increased by 2.05 kg less (95% *CI* [−3.40 to −0.70]) than participants in control conditions. One study was synthesised narratively and indicated a direction in favour of the intervention.

#### Physical activity

Of the 36 studies measuring changes in physical activity levels, 11 studies that used the same tool (IPAQ total MET minutes/week) were included in a meta-analysis (10 SMI only; 1 non-SMI). Meta-analysis indicated interventions had a positive effect, an increase of 226.82 (95% CI [22.03 to 431.61]) metabolic minutes per week at the end of intervention when compared to control groups. Of the 25 studies in the narrative synthesis that examined physical activity using other outcome measures, 18 studies indicated a direction in favour of the intervention, 5 in favour of control and 2 in neither direction.

#### Diet

Of the 14 studies measuring changes in diet, 3 were included in a meta-analysis for the measure of fruit serves (all SMI only). This analysis did not indicate evidence of an effect, with intervention participants increasing consumption by 0.01 (95% *CI* [−0.29 to 0.31]) serves per day compared to control conditions. Three studies were included in a meta-analysis for the measure of vegetable serves (all SMI only), which found interventions had a positive effect, with intervention participants increasing consumption by 0.51 (95% CI [0.11 to 0.92]) serves per day compared to control conditions. Of the 13 studies synthesised narratively across other diet measures, 11 studies indicated a direction in favour of the intervention and 1 in favour of control and 1 in neither direction.

### Secondary outcomes

#### BMI

Of the 34 studies that measured change in BMI, 33 were included in meta-analysis (30 SMI only; 2 non-SMI; 1 mixed). Meta-analysis of data indicated a positive effect of interventions to reduce BMI, with average BMI reduction across studies −0.48 units (95% CI [−0.70 to −0.26]) when compared to control conditions. One study was synthesised narratively and indicated a direction in favour of the intervention.

#### Waist circumference

Of the 23 studies that measured change in waist circumference, 19 were included in a meta-analysis (all SMI only). Meta-analysis indicated a positive effect of interventions, with average waist circumference reduction across studies −0.87cms (95% CI [−1.60 to −0.14]) when compared to control conditions. Of the 4 studies synthesised narratively, 2 studies indicated a direction in favour of the intervention and 2 in neither direction.

#### Sedentary behaviour

The 3 studies that measured change in sedentary behaviour were not amenable to meta-analysis, and all were included as narrative synthesis. Two studies indicated a direction in favour of the control group and 1 in neither direction for the sedentary behaviour outcomes examined.

#### Mental health

Standardised mean differences (SMDs) were used to pool data across studies using different measurement tools for mental health outcomes (supplementary Table 4). Of the 25 studies measuring changes in mental health, 14 were included in meta-analysis for the outcomes of depression, anxiety and/or severity of psychological symptoms, and 18 were included in the narrative syntheses for other mental health measures.

##### Depression

Twelve studies measuring depression (using 6 comparable depression scales/tools) were included in meta-analysis (8 SMI only; 4 non-SMI). The analysis did not indicate evidence of an effect. The pooled effect of interventions on depression as a standardised mean difference (SMD) was −0.03 (95% *CI* −0.14 to 0.08) when compared to control conditions. Two studies were synthesised narratively, and both indicated a direction in favour of the intervention.

##### Anxiety

Five studies measuring anxiety (using 3 comparable anxiety scales/tools) were included in meta-analysis (3 SMI only; 2 non-SMI). The analysis did not indicate evidence of an effect. The pooled effect of interventions on anxiety as a standardised mean difference (SMD) was −0.49 (95% *CI* −1.15 to 0.16) when compared to control conditions. Two studies were synthesised narratively, and both indicated a direction in favour of the intervention.

##### Severity of psychological symptoms

Four studies measuring severity of psychological symptoms (using 2 comparable scales/tools) were included in meta-analysis (3 SMI only; 1 non-SMI). The analysis did not indicate evidence of an effect. The pooled effect of interventions on severity of psychological symptoms as a standardised mean difference (SMD) was 0.72 (95% *CI* −0.05 to 1.50) when compared to control conditions.

##### Other mental health measures/variables

Of the remaining 14 studies synthesised narratively, 9 indicated a direction in favour of the intervention and 5 in favour of control for the mental health outcomes examined.

### Assessment of methodological quality

#### Risk of bias in included studies and quality of evidence

**Fig. 2:**
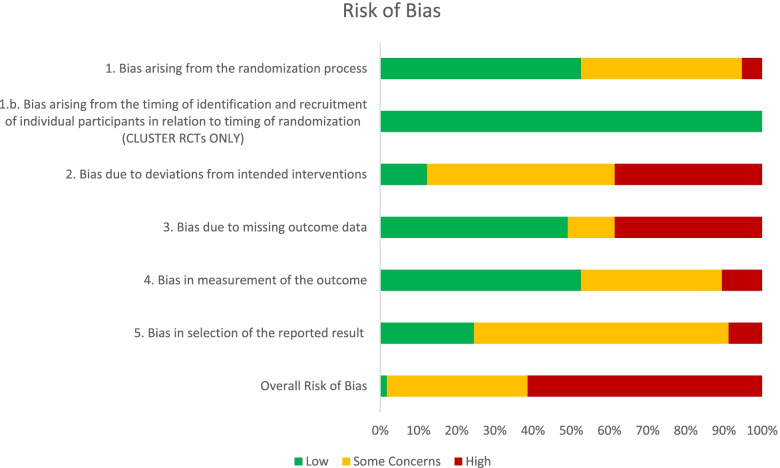
Risk of bias in included studies

Assessment of risk of bias is shown in Fig. 2. Of the 52 individually randomised controlled studies, 32 (62%) were rated as high risk for bias overall, while 3 of 5 cluster-randomised studies were rated as high overall. The quality of evidence (GRADE) for weight outcomes was downgraded to “moderate” due to considerable statistical heterogeneity, the quality of evidence for physical activity was downgraded to “moderate” due to lack of blinding of participants, and the quality of evidence for diet was downgraded to “low” due to lack of blinding of participants and imprecise results.

## Discussion

This is the first review to synthesise evidence from randomised controlled trials on the effectiveness of lifestyle interventions delivered in a community or outpatient setting for people with a mental health condition to improve weight, physical activity and/or diet. The review represents a thorough consideration of evidence across a comprehensive range of anthropometric, physical activity and diet-related outcomes. Meta-analyses revealed statistically significant improvements in primary outcomes of weight; physical activity, as measured by IPAQ MET minutes; and diet, in the form of increased vegetable consumption but no significant improvement for fruit consumption. Narrative synthesis through vote counting for studies not included in meta-analyses for the primary outcomes indicated a greater number of studies reporting improvements in the intervention group than for the control group or neither group. For secondary review outcomes, meta-analyses indicated statistically significant improvements in BMI and waist circumference, but not for anxiety, depression or severity of psychological symptoms. Narrative synthesis for sedentary behaviour did not support an effect. The majority of studies included only participants with SMI. Risk-of-bias assessment found the majority of studies were high risk or had some concerns overall, with bias most commonly pertaining to missing outcome data and deviations from interventions. Quality of evidence for weight and physical activity was moderate, while quality of evidence for diet was low

Anthropometric findings from the current review show some consistency with meta-analysis findings from previous systematic reviews of lifestyle interventions in mental health samples with SMI, concluding modest but significant effects on weight [20–23], BMI [20, 24–26] and waist circumference [23, 25]. In general, the magnitude of changes in the anthropometric variables was smaller in the current review than in other recent reviews focussing on people with mental health conditions. For example, the mean weight loss of 1.42 kg found in the present review is slightly less than the 2.01 kg mean loss reported in a recent review in which weight outcomes were reported in a similar manner [20]. Our review inclusion criteria, which encompassed a broader range of mental health conditions beyond those considered to be SMI, contributed very few studies to the meta-analyses of anthropometric outcomes — the results largely represent those contributed by studies of SMI participants. It is important to acknowledge that additional difficulties in achieving changes to anthropometric measures may be experienced by people with a mental health condition who are taking psychoactive medications, which often have weight gain as a side effect [117, 118]. Our meta-analysis based on studies that included only those on such medications, with a focus on weight maintenance, showed that intervention participants increased by 2.05 kg less than participants in control conditions suggesting interventions can be of value for these groups. Future research should consider whether lifestyle interventions are equally as effective for those taking such medications compared to those not and, if not, how the impact of such medications may best be minimised to reduce the impact on physical health [2].

This is the first review incorporating meta-analysis to examine the effects of lifestyle interventions for people with a mental health condition on physical activity. While a majority of included studies (*n* = 34) reported on a physical activity outcome variable, the diversity in measurement tools used, including several instances of a tool being used in only one study, meant that more than half of these were not included in meta-analyses, and only one of the included studies was not only participants with SMI [119, 120]. Based on studies using IPAQ MET minutes, findings suggest that lifestyle interventions lead to significant improvements in number of MET minutes achieved per week among intervention participants, with average increase in physical activity of 266 MET minutes aligning with more than half the recommended MET minutes per week for physical activity [121]. The narrative vote counting based on direction of effect of studies measuring physical activity in other formats also supports the finding of a positive effect. The current review reports limited evidence on sedentary behaviour with no evidence of intervention effect for this outcome. Our review adds to the evidence base on the impact of lifestyle interventions on physical activity and sedentary behaviour for those with a mental health condition, previously summarised in a narrative review of studies involving those with SMI, which concluded there was inconsistent evidence to show interventions can be effective in changing these outcomes for this group, and noted the lack of studies focussed on sedentary behaviour [27].

This is the first review to synthesise evidence for the impact of lifestyle interventions for people with a mental health condition on diet. Fourteen studies assessed improvements in diet, with the most commonly measured dietary outcomes relating to fruit and vegetable intake. Three studies contributed to meta-analyses for both fruit serves (showing no effect) and vegetable serves (showing a small effect of about half a serve daily). Of the remaining studies which contributed to narrative synthesis, the majority incorporated other validated measures of dietary variables. The seven studies that reported other fruit and/or vegetable variables, and the six studies reporting other dietary variables (e.g. adherence to a Mediterranean diet), indicated a direction of effect favourable to the intervention groups. Variability in measurement of dietary variables may be partly attributable to differences in what the lifestyle programmes were aiming to achieve and perhaps to global diversity in diets. Consistent measurement of variables including fruit and vegetable intake would assist in future syntheses. Trials amongst the general population suggest combined fruit and vegetable intake may be increased by up to 1.18 serves following the receipt of dietary advice, a larger effect than suggested in the current review [16]. Such findings and the small number of studies in our review suitable for meta-analysis highlight the need for further studies assessing the potential for lifestyle interventions to facilitate improvements in diet amongst people with a mental health condition.

Results regarding the effect of lifestyle interventions on measures of mental health were mixed. Meta-analysis for specific mental health outcome variables was possible only for anxiety and depression. No significant effects were found for either depression or anxiety nor for severity of psychological symptoms. Narrative synthesis across a range of other mental health measures indicated variable findings. It is possible that the inclusion of participants with more SMI (such as schizophrenia) in a majority of studies, for whom change in the lifestyle factors examined may be especially challenging, could have contributed to the variability in findings.

As noted above, it is important to acknowledge that the majority of studies in this review included only participants considered to have an SMI. While this review was designed to be inclusive of all mental health conditions and highlight the current research landscape of lifestyle interventions for this population, the findings cannot be considered reflective of all mental health conditions due to the limited number of trials in non-SMI groups. Given that SMI makes up a small proportion of people with mental health conditions, it is recommended that additional research into lifestyle interventions for people with mental health conditions other than SMI is conducted. The clinical significance of changes achieved through lifestyle interventions varies by outcome measure. It has been suggested that the scale of changes in outcomes such as weight and BMI found in the current review and previous reviews may be of questionable clinical significance [20, 26]. While many studies reported weight loss or reduction in BMI, few studies reported the proportion of participants achieving 5% weight loss, an amount deemed clinically significant for reducing health risks [122], which did show significant effect in the current review. While it has been suggested that even small improvements to physical activity are associated with a reduction in health risk in the general population [123], the increase in fruit and vegetable consumption required to lead to meaningful change is unknown. Further research providing a benchmark for clinically significant change across health measures in this population would be helpful in the evaluation of future interventions.

Other limitations to the evidence base and review findings should also be noted. Planned subgroup analyses, by intervention delivery mode, intervention topic focus and participant mental health condition or groupings of condition (e.g. SMI vs other), were not able to be conducted due to insufficient studies or insufficient study detail required for grouping. Few studies explored interventions delivered via modalities other than face to face such as online, or via telephone, which some research has reported as being feasible within this population [91, 124, 125]. Across all outcome measures, there was a high risk of bias across the majority of included studies. Heterogeneity within meta-analyses for all continuous anthropometric outcomes was high. Analyses do not account for variability in intervention length, which ranged from 1 week to 12 months; however, 48 of the 57 interventions included in this review were of at least 12-week duration. The present analyses do not allow examination of maintenance effects. Analyses are based on immediate post-intervention outcomes to maximise the chance of capturing intervention effects and as many studies reported only baseline and immediate end of intervention outcome measures.

## Conclusion

This review synthesised evidence from randomised controlled trials on efficacy of lifestyle interventions in improving several health measures and behaviours among people with a mental health condition. Findings based on moderate or low-quality evidence suggest that such interventions are effective in creating statistically significant but small positive changes to weight, BMI, waist circumference, physical activity and vegetable consumption, but not fruit consumption. Variability in measurement tools or insufficient reported data impacted capacity to meta-analyse some outcomes. Vote counting indicated a direction of effect in favour of intervention groups for studies assessing physical activity and diet that were not included in meta- analyses but did not support an effect for sedentary behaviour or mental health measures. The findings support the value of lifestyle interventions to improve health risks in people with a mental health condition but point to the need for further high-quality trials and a consideration of the clinical significance of findings. Improving consistency in data collection and reporting across intervention trials may contribute to higher quality evidence and assist in determining factors that contribute to intervention effectiveness. Furthermore, while the current review extends on previous research by including client samples beyond SMI, this extension did not contribute a large number of studies. Research which allows for examination of the efficacy of lifestyle interventions for specific mental health conditions would advance the field.

## Supplementary Information

**Additional file 1: Supplementary Table 1.** Search Strategy (PSYCINFO). **Supplementary Table 2.** Approaches to calculate required data for meta-analyses when not presented in published studies. **Supplementary Table 3.** Summary of narrative synthesis. **Supplementary Figure 1.** Meta-analysis for weight loss (kgs). **Supplementary Figure 2.** Meta-analysis for weight loss (5% body weight loss). **Supplementary Figure 3.** Meta-analysis for weight maintenance (kgs). **Supplementary Figure 4.** Meta-analysis for physical activity (IPAQ - Met Minutes). **Supplementary Figure 5.** Meta-analysis for diet (fruit serves daily). **Supplementary Figure 6.** Meta-analysis for diet (vegetable serves daily). **Supplementary Figure 7.** Meta-analysis for BMI. **Supplementary Figure 8.** Meta-analysis for waist circumference (cms). **Supplementary Table 4.** Pooled measures for mental health. **Supplementary Figure 9.** Meta-analysis for Depression (SMD). **Supplementary Figure 10.** Meta-analysis for Anxiety (SMD). **Supplementary Figure 11.** Meta-analysis for Severity of Psychological Symptoms (SMD).

## Data Availability

All data generated or analysed during this study are included in this published article.
