# Supplementary material for: Systematic review of lifestyle interventions to improve weight, physical activity and diet among people with a mental health condition
Source: Syst Rev. 2022 Sep 9;11:198. doi: 10.1186/s13643-022-02067-3 (PMC9462072; doi:10.1186/s13643-022-02067-3)

**SUPPLEMENTARY MATERIALS**

Supplementary Table 1. Search Strategy (PSYCINFO)

| **#** | **Searches** | **Results** |
| --- | --- | --- |
|  | HEALTH BEHAVIOURS |  |
| 1 | eating behaviour/ or food preference/ or diets/ or nutrition/ | 23388 |
| 2 | (fruit* or vegetable*).tw. | 18380 |
| 3 | exercise/ or physical activity/ or sedentary behaviour/ | 34165 |
| 4 | (physical activit* or exercise or physical fitness or physical inactivit* or sedentary).tw. | 75303 |
| 5 | obesity/ or overweight/ or body weight/ or weight control/ or weight loss/ | 36267 |
| 6 | risk factors/ or lifestyle/ or health behaviour/ | 81621 |
| 7 | chronic illness/ or disease management/ or cardiovascular disorders/ | 25769 |
| 8 | (cardiovascular adj3 (disease* or risk*)).tw,kw. |  |
| 9 | 1 or 2 or 3 or 4 or 5 or 6 or 7 OR 8 | 230296 |
|  |  |  |
|  | MENTAL HEALTH POPULATION |  |
| 10 | mental disorders/ or mental health/ or chronic mental illness/ | 132705 |
| 11 | (mental health or mental illness* or psychiatric or mental disorder*).mp. | 442169 |
| 12 | 9 or 10 | 442169 |
|  |  |  |
|  | INTERVENTION |  |
| 13 | interventions/ or health education/ or health promotion/ | 33487 |
| 14 | ((health* or lifestyle* or life style*) adj5 (intervention* or therap* or treatment* or program* or promot*)).tw. | 96926 |
| 15 | 12 or 13 | 111882 |
|  |  |  |
|  | STUDY DESIGN |  |
| 16 | random*.tw. | 184796 |
| 17 | trial.tw. | 98308 |
| 18 | groups.tw. | 475952 |
| 19 | 15 or 16 or 17 or 18 | 662822 |
|  |  |  |
|  | COMBINED |  |
| 20 | 8 and 11 and 14 and 19 | 764 |
| 21 | limit 20 to yr="1998 - 2020" | 741 |

| Supplementary Table 2. Approaches to calculate required data for meta-analyses when not presented in published studies | |
| --- | --- |
| Annotations next to corresponding studies in each of the meta-analyses | [a] – Standard Deviations carried across from baseline to post scores. Assumption that the variability in post would be similar to the variability in pre, therefore the available standard deviation at baseline was used for the post intervention score.  [b] - Missing Standard Deviations at pre and/or post were obtained from the provided confidence interval for pre and/or post scores.  [c] - Calculated standard deviations for pre and post scores were used (with correlation coefficients) to determine the standard deviation for the mean change score.  [d] - Missing standard deviations for change scores were obtained from the provided confidence interval for change scores.  [e] - Raw data (mean differences and their standard deviations) were adjusted to accurately reflect the modelled Effect Sizes reported in published data. Means and Standard deviations were generated in order to match the reported modelled effect size reported in the study. |
| Correlation coefficients | Where standard deviations for change scores were not reported, but were reported pre and post intervention measurements, to reduce the width of the confidence interval for the effect size for the change, we used an estimate of the correlation between pre and post scores. However, as many studies did not report this information, plausible values were obtained from those studies in the review that did provide it and also a sensitivity analysis whereby the value of the correlation coefficient was varied. Based on those two assessments, the following correlation coefficients were used: 0.95 (weight loss), 0.95 (BMI), 0.90 (waist circumference). Correlation coefficients of 0.00 were used for the meta-analyses on mental health (depression, anxiety, severity of psychological symptoms) and physical activity (IPAQ Met minutes). All other meta-analysis did not require correlation coefficients. |
| Sensitivity Analyses | Weight |
|  | BMI |
|  | Waist Circumference |

Supplementary Table 3. Summary of narrative synthesis

| **Measurement** | **Study** | **Available data^a^** | **Direction^b^** |
| --- | --- | --- | --- |
| **Weight (loss)** |  |  |  |
| % participants maintained or lost weight | Holt 2019 | *Odds ratio (control group as reference)*  Intervention group (n=178)  Control group (n=180)  1.35 (0.85 to 2.14)  Non-significant | 1 |
|  | Daumit 2013 | *Proportion of sample*  Intervention group (n=142): 62.6%,  Control group (n=146): 51.1%  *p* = 0.05 | 1 |
| % participants that lost 7% of body weight | Erickson 2016 | *Proportion of sample*  Intervention group (n=60): 22%  Control group (n=48): 12%  *p* = 0.21 | 1 |
| % participants that lost more than 10 lbs | Brown 2011 | *Proportion of sample*  Intervention group (n=47): 32%  Control group (n=42): 10%  Significance not reported | 1 |
| Weight loss (kg) | Erickson 2017 | *Change at end of intervention period* Intervention group (n=53)  Control group (n=50)  Both groups lost weight compared to baseline, though differences between groups at 1 year were not statistically significant | - |
|  | Brown 2006 | *Mean change*  Intervention group (n=7): -0.4  Control group (n=10): 1.11  Non-Significant | 1 |
|  | Brown 2009 | *Mean change*  Intervention group (n=15): -0.8  Control group (n=11): 1.8  *p* = 0.3 | 1 |
|  | Muralidharan 2018 | *Mean change*  Intervention group (n=71): -2.8  Control group (n=88): 0.3  Significance not reported | 1 |
| Weight loss (lbs) | Jean-Baptiste | *Mean change*  Intervention group (n=8): -6.4  Control group (n=6): 5.9  *p* = 0.26 | 1 |
| **Weight (maintenance)** |  |  |  |
| Increase of more than 7% | Alvarez-Jimenez 2006 | *Proportion of sample*  Intervention group (n=28): 39%  Control group (n=33): 79%  *p*<.002 | 1 |
| **Physical activity** |  |  |  |
| *Accelerometer / Pedometer* |  |  |  |
| Total activity (square root of minutes) - Accelerometer | McKibbin 2006 | *Mean (SD) at end of intervention period*  Intervention group (n = 28): 22.6 (16.8)  Control group (n = 29): 13.6 (15.0)  Non-significant | 1 |
| Clinically sig. physical activity (>=30mins MVPA per day) - Accelerometer | Petzold 2018 | *Proportion at end of intervention*  Intervention group (n=43): 76.7%  Control group (n=40): 27%  Significance not reported | 1 |
| Moderate plus vigorous physical activity – (minutes) Accelerometer | Holt 2019 | *Mean difference (95% CI)*  Intervention group (n=178)  Control group (n=180)  1.5 (-2.5 to 5.5)  Non-significant | 1 |
| Minutes of moderate plus vigorous physical activity [MVPA] (weekly) - Accelerometer | Pentecost 2015 | *Mean (SD) at end of intervention period*  Intervention group (n = 15): 360 (186)  Control group (n = 13): 339 (170)  Significance not reported | 1 |
| Mean steps (daily) - Pedometer | Forsberg 2008 | *Mean (SD) at end of intervention period*  Intervention group (n = 9): 5586 (33.13)  Control group (n = 8): 4655 (32.04)  Non-significant | 1 |
| *Validated self-report measure* |  |  |  |
| Moderate physical activity (# sessions of per week) - Godin Leisure Time Questionnaire | Brown 2009 | *Mean change*  Intervention group (n=15): 0.5  Control group (n=11): 0  *p* = 0.02 | 1 |
| Moderate physical activity (# sessions of per week) - Godin Leisure Time Questionnaire | Brown 2006 | *Mean change*  Intervention group (n=7): 2.4  Control group (n=10): 0.2  *p* = 0.04 | 1 |
| Mild physical activity (# sessions of per week) - Godin Leisure Time Questionnaire | Daley 2008 | *Mean difference (95% CI)*  Intervention group (n=16)  Control group (n=15)  0.7 (–0.8 to 2.3)  Non-significant | 1 |
| Total score - Godin Leisure Time Exercise Questionnaire | Ratliff 2012 | *Mean change (SD) at end of intervention period*  Control* group (n=8): -5.5 (35)  Intervention* group (n=8): 12.1 (19.4)  *p*=.55  **Within subjects. Control group became intervention group [CM-behaviour] after initial 8 week period.* | 1 |
| Total vigorous activity score (log of MET min) - IPAQ | Bartels 2013 | *Mean (SD) at end of intervention period*  Intervention group (n = 67): 393.7 (1048.8)  Control group (n = 66): 484.3 (1992.6)  *p* = .001 | 1 |
| Total vigorous activity score (log of MET min) - IPAQ | Bartels 2015 | *Mean (SD) at end of intervention period*  Intervention group (n = 104): 379.8 (890.8)  Control group (n = 106): 433.7 (1080.1)  *p* = .006 | 1 |
| Total Metabolic minutes (per week) – IPAQ | Daley 2015 | *Mean difference in adjusted medians (95% CI)*  Intervention group (n=33)  Control group (n=40):  -546 (-1194 to 102)  *p* = .10 | 0 |
| Walking time (minutes per week) - IPAQ | Baker 2018 | *Mean change (95% CI)*  Intervention group (n=71): 96.3 (-113 to 306)  Control group (n=68): -38.9 (-179 to 101)  *p* = .079 | 1 |
| Walking time (minutes per day) - IPAQ | Lee 2014 | *Mean (SD) at end of intervention period*  Intervention group (n = 8): 48.77 (58.07)  Control group (n = 8): 93.61 (66.83)  Significance not reported | 0 |
| Walking time (MET minutes per week) - IPAQ | Muralidharan 2018 | *Mean (SD) at end of intervention period*  Intervention group (n = 76): 748 (979)  Control group (n = 74): 545 (710)  Comparison group 2 (n=81): 500 (493)  p = .076*  **Difference in intervention & control only* | 1 |
| Moderate-vigorous physical activity (hours/week) – Physical Activity Scale | Speyer 2015 | *Mean (SD) at end of intervention period*  Intervention group (n = 120): 2.5 (4.0)  Control group (n = 127): 2.5 (4.0)  Comparison group 2 (n=120): 3.1 (4.4)  *p* = .43 | - |
| Total time in physical activity (min p/week) – Active Australia Survey | Forsyth 2015 | *Mean change (percentage improvement)*  Intervention group (n = 31): 102.9 (31.%)  Control group (n = 31): 114.0 (35.8%)  *p* = .73 | 0 |
| Moderate or vigorous physical activity (Minutes per week) - Behavioral Risk Factor Surveillance System | Druss 2010 | *Mean (SD) at end of intervention period*  Intervention group (n = 37): 191 (278)  Control group (n = 28): 152 (249)  *p* = .40 | 1 |
| *Other* |  |  |  |
| Walking time (minutes per month) - monitored by study personnel | Beebe 2010 | *Mean (SD) at end of intervention period*  Intervention group (n = 37): 116.89 (117.3)  Control group (n = 42): 78.83 (106.4)  *p* = .671 | 1 |
| Physical Activity Habits | Gyllensten 2017 | *Proportion at end of intervention*  Intervention group (n=45)  Sedative: 6  Some exercise: 12  Regular exercise: 8  Training: 7  Missing: 12  Control group (n=30)  Sedative: 3  Some exercise: 8  Regular exercise: 5  Training: 5  Missing: 13  Non-significant | - |
| Exercise per week (minutes) – *Two-item measure* | Abrantes 2017 | *Mean (SD) at end of intervention period*  Intervention group (n = 21): 133.1 (68.14)  Control group (n = 27): 96.3 (117.75)  *p* = .028 | 1 |
| Meeting 1000 MET minutes per week (deemed physically active) – *New scale devised for study* | Chalder 2012 | *Odds ratio (control group as reference)*  Combined (n=174)  1.86 (1.0 to 3.46)  Significance not reported | 1 |
| # of days per week engaging in >30mins moderate physical activity - *Tool not reported* | Goldberg 2013 | *Mean (SD) at end of intervention period*  Intervention group (n = 30): 3.6 (2.7)  Control group (n = 41): 3.9 (2.7)  *p* = .396 | 0 |
| Minutes of exercise (per week) - *Lifestyle habits questionnaire* | Erickson 2017 | *Mean change*  Intervention group (n=53): 33  Control group (n=50): 9  *P =* 0.41 | 1 |
| Engaging in moderate physical activity (like brisk walking) for at least 30 min on at least 5 days a week - *Tool not reported* | Bonfioli 2018 | *Proportion at end of intervention*  Intervention group (n=169): 87 (51%)  Control group (n=156): 55 (35%)  Significance not reported | 0 |
| **Diet** |  |  |  |
| *Fruit and/or vegetables* |  |  |  |
| Self-report Fruit and/or Veg (values combined) (Daily serves) | Baker 2018 | *Mean change (95% CI)*  Intervention group (n=71): -0.1 (-0.8 to 0.7)  Control group (n=68): -0.4 (-1.1 to 0.3)  *p* = .330 | 1 |
| Scottish Health Survey Fruit and/or Veg (values combined) (weekly serves) | McCreadie 2005 | *Mean (SD) at end of intervention period*  Intervention group (n = 32): 30 (19)  Control group (n = 27): 18 (15)  Comparison group 2 (n=37): 31 (24)  Significant (*p-*value not reported) | 1 |
| DINE Questionnaire  Fruit OR Vegetable  (daily serves) | Brown 2009 | *Mean change*  Intervention group (n=15): 1.2  Control group (n=11): 0.0  *p* = 0.05 | 1 |
| Grams of Fruit p/week | Speyer 2016 | *Mean (SD) at end of intervention period*  Intervention group (n = 120): 393.1 (268.5)  Control group (n = 127): 421.4 (258.1)  Comparison group 2 (n=120): 439.8 (270.7)  *p* = .31 | 0 |
| Insufficient fruit or vegetable consumption over the past year | Goldberg 2013 | *Proportion at end of intervention*  Intervention group (n=30): 30%  Control group (n=41): 37%  *P* = .678 | 1 |
| Meeting WHO dietary guidelines (Consuming at least five servings of fruit and/or vegetables a day (400–500 g daily)) | Bonfioli 2018 | *Proportion at end of intervention*  Intervention group (n=169): 2 (1%)  Control group (n=156): 0 (0%)  Significance not reported. | 1 |
| Consuming >= 5 portions of fruit and vegetables per day | Petzold 2018 | *Proportion at end of intervention*  Intervention group (n=43): 3 (7%)  Control group* (n=40): 15 (37%)  Significance not reported  **Aim of study to increase physical activity. As attention control, while intervention group content focused on physical activity, control group focus on diet* | - |
| *Other* |  |  |  |
| ModiMedDiet score | Jacka 2017 | *Between groups difference in change from baseline to intervention (n=56)*  -20.7 (-20.7 -12.1)  *p* = <.001 | 1 |
| Diet History Questionnaire (Australian Healthy Eating Index) | Forsyth 2015 | *Mean change (percentage improvement)*  Intervention group (n = 32): 2.4 (5.5%)  Control group (n = 31): 1.5 (3.5%)  *p* = .28 | 1 |
| Block Fat-Sugar-Fruit-Vegetable Screener | Druss 2018 | *Mean (SD) at end of intervention period*  Intervention group (n=198): 10.2 (5.6)  Control group (n=202): 9.4 (5.3)  *p* = .72 | 1 |
| QUMDA score | Bersani 2017 | *Mean (SD) at end of intervention period*  Intervention group (n=16): 8.75 (1.81)  Control group (n=16): 6.81 (1.55)  *p* = .005 | 1 |
| Food Frequency Questionnaire score | Lovell 2014 | *Mean change (SD)*  Intervention group (n=48): 5.0 (11.1)  Control group (n=90): 0.2 (8.0)  *p =* .018 | 1 |
| PREDIMED score | Masa-Font 2015 | *Mean difference (95% CI)*  Intervention group (n=169):  Control group (n=163):  0.25 (-0.18 to 0.68)  *p = .*256 | 1 |
| **BMI** |  |  |  |
|  | Brown 2006 | *Mean change*  Intervention group (n=7): -0.02  Control group (n=10): 0.41  *p =* .02 | 1 |
| **Waist Circumference** |  |  |  |
| Elevated WC | Goldberg 2013 | *Proportion at end of intervention*  Intervention group (n=30): 86%  Control group (n=41): 77%  p = .678 | 1 |
| Waist circumference (cm) | Erickson 2017 | *Mean change*  Intervention group (n=53) -1.04  Control group (n=50) 0.25  p < 0.001 | 1 |
|  | Ratliff 2012 | *Mean change (SD) at end of intervention period*  Control* group (n=8): 3.4 (7.0)  Intervention* group (n=8): -3.5 (5.6)  *p*=.39  **Within subjects. Control group became intervention group [CM-behaviour] after initial 8 week period.* | - |
|  | Forsberg 2008 | Men only  *Mean (SD) at end of intervention period*  Intervention group (n = 12): 110 (13)  Control group (n = 9): 113 (14)  Women only  Intervention group (n = 9): 108 (27)  Control group (n = 4): 83 (16)  Not significant | - |
| **Sedentary Behaviour** |  |  |  |
| Sitting time (mins per week) | Baker 2018 | *Mean change (95% CI)*  Intervention group (n=71): -27.3 (-628 to 574)  Control group (n=68): -210.6 (-784 to 363)  *p* = .574 | 0 |
| Accelerometer measured average minutes of sedentary activity daily | Pentecost 2015 | *Mean (SD) at end of intervention period*  Intervention group (n = 15): 373 (165)  Control group (n = 13): 393 (177)  *p* = NR | 0 |
| Time spent sedentary (hrs per day) | Speyer 2016 | *Mean (SD) at end of intervention period*  Intervention group (n = 120): 9.9 (3.6)  Control group (n = 127): 9.9 (3.5)  Comparison group 2 (n=120): 10.5 (3.4)  *p* = .36 | - |
| **Mental health** |  |  |  |
| *Anxiety* |  |  |  |
| Hospital Anxiety and Depression Scale (HADS-A) | Brown 2006 | *Mean change*  Intervention group (n=15): -0.02  Control group (n=13): 0.41  *p* = 0.19 | 1 |
|  | Brown 2009 | *Mean change*  Intervention group (n=15): -0.1  Control group (n=11): 0.3  *p* = 0.5 | 1 |
| *Depression* |  |  |  |
| Hospital Anxiety and Depression Scale (HADS-D) | Brown 2006 | *Mean change*  Intervention group (n=15): -1.1  Control group (n=13): 1.3  *p* = 0.08 | 1 |
|  | Brown 2009 | *Mean change*  Intervention group (n=15): -0.7  Control group (n=11): 0.2  *p* = 0.7 | 1 |
| *Mental Health Symptomology* |  |  |  |
| Global Assessment of Functioning | Gaughran 2017 | *Mean difference (95% CI)*  Intervention group (n = 126)  Control group (n = 130)  -0.7 (–3.44 to 2.03)  *p* = 0.62 | 1 |
|  | Mauri 2008 | *Mean change (SD)*  Intervention group (n=21): 0.9 (2.8)  Control group (n=24): 2.6 (6.3)  Non-significant | 1 |
| Symptom Checklist-90-R (Global Score) | Skrinar 2005 | *Mean Change*  Intervention group (n=9): 19.27  Control group (n=11): -.18  *p* = 0.31 | 0 |
| Clinical Global Impressions Scale | Masa-Font 2015 | *Mean difference (95% CI)*  Intervention group (n = 168)  Control group (n = 161)  -0.11 (–0.31 to 0.10)  *p* = 0.312 | 1 |
| Clinical Global Impressions Scale – Severity of Illness | Goracci 2016 | *Mean change (SD)*  Intervention group (n=81): -0.32 (1.08)  Control group (n=79): -0.19 (1.18)  *p* = 0.67 | 1 |
| *Post Natal Depression* |  |  |  |
| Edinburgh Postnatal Depression Scale | Daley 2015 | *Adjusted Mean difference (95% CI)*  Intervention group (n = 43)  Control group (n = 42)  -2.26 (–4.36 to -0.16)  *p* = 0.035 | 1 |
|  | Daley 2008 | *Mean difference (95% CI)*  1.2 (–5.2 to 2.8)  Non-significant | 0 |
| *Post Traumatic Stress Disorder* |  |  |  |
| PTSD CheckList – Civilian Version | Kilbourne 2017 | *Mean difference (95% CI)*  Intervention group (n = 124)  Control group (n = 121)  -0.59 (–3.92 to 2.74)  *p* = 0.72 | 1 |
| *Obsessive-Compulsive Disorder* |  |  |  |
| Yale-Brown Obsessive Compulsive Scale | Abrantes 2017 | *Mean (SD) at end of intervention period*  Intervention group (n = 23): 19.4 (6.4)  Control group (n = 26): 19.9 (6.43)  Effect Size −0.17  Not Significant | 1 |
| *Schizophrenia* |  |  |  |
| Positive and Negative Syndrome Scale | Gaughran 2017 | *Mean difference (95% CI)*  Intervention group (n = 126)  Control group (n = 130)  1.37 (-1.23 to 3.96)  *p* = 0.3 | 0 |
| Scale for the Assessment of Negative Symptoms | Speyer 2016 | *Mean (SD) at end of intervention period*  Intervention group (n = 120): 2.1 (1.2)  Control group (n = 127): 2.0 (1.2)  Comparison group 2 (n=120): 2.0 (1.2)  *p* = .52 | 0 |
| Scale for the Assessment of Positive Symptoms | Speyer 2016 | *Mean (SD) at end of intervention period*  Intervention group (n = 120): 1.7 (1.6)  Control group (n = 127): 1.8 (1.6)  Comparison group 2 (n=120): 1.7 (1.6)  *p* = .29 | 1 |
| *Other* |  |  |  |
| Overall BASIS-24 Symptoms score | Kilbourne 2017 | *Mean difference (95% CI)*  Intervention group (n = 124)  Control group (n = 121)  -0.05 (–0.21 to 0.11)  *p* = 0.55 | 1 |
| Mental Health Inventory | Marzolini 2009 | *Mean (SD) at end of intervention period*  Intervention group (n = 4): 65.5 (9)  Control group (n = 6): 58.1 (5.5)  *p* = .33 | 0 |

^a^ The ‘available data’ column indicates the data that were directly reported, in terms of: effect estimate, direction of effect, confidence interval, precise p-value, or statement regarding statistical significance (either statistically significant or not).

^b^ Direction of effect (based on greater change or better health status): 1 indicates greater improvements in intervention group, 0 indicates greater improvements in control group, - indicates neither direction

Supplementary Figure 1. Meta-analysis for weight loss (kgs)

**
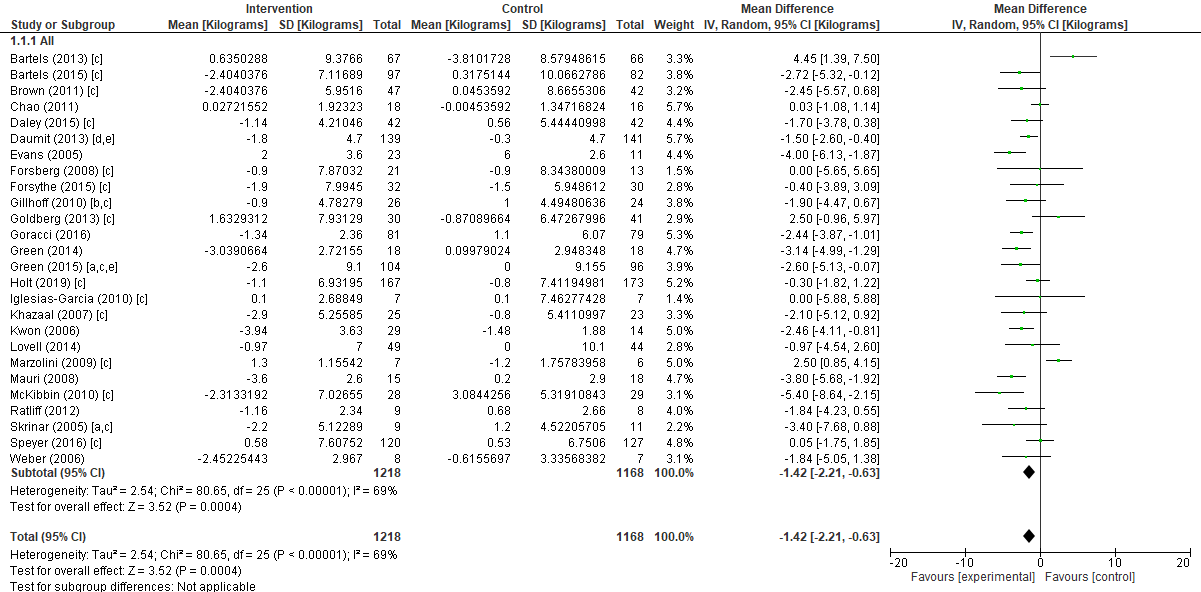
**

Supplementary Figure 2 Meta-analysis for weight loss (5% body weight loss)


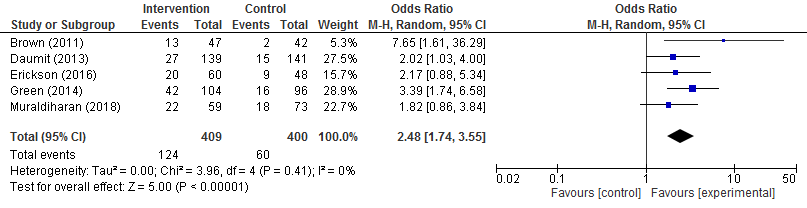


Supplementary Figure 3. Meta-analysis for weight maintenance (kgs)

**
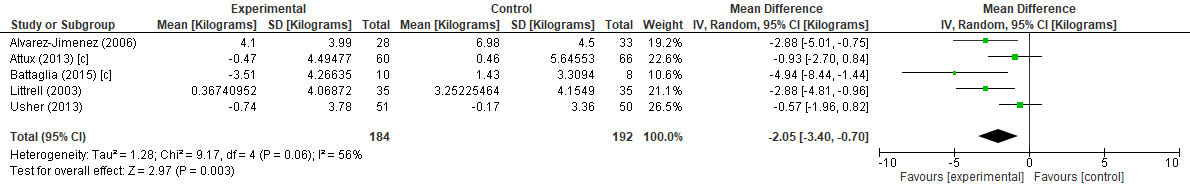
**

Supplementary Figure 4. Meta-analysis for physical activity (IPAQ - Met Minutes)


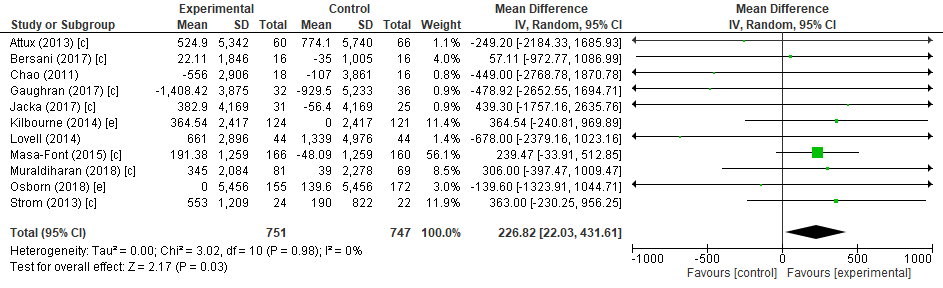


Supplementary Figure 5. Meta-analysis for diet (fruit serves daily)

**
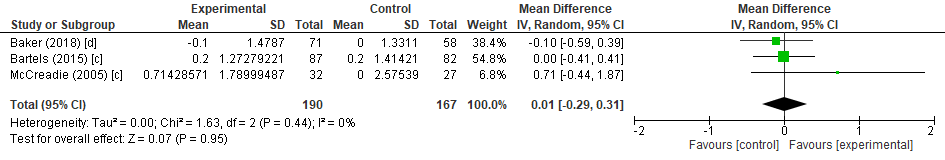
**

Supplementary Figure 6. Meta-analysis for diet (vegetable serves daily)


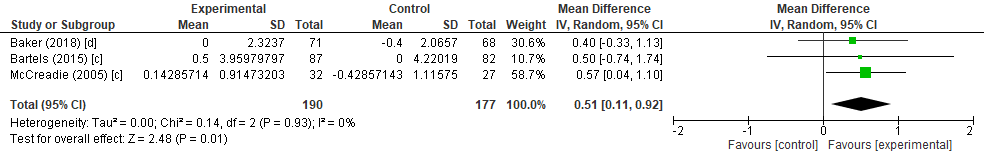


Supplementary Figure 7. Meta-analysis for BMI


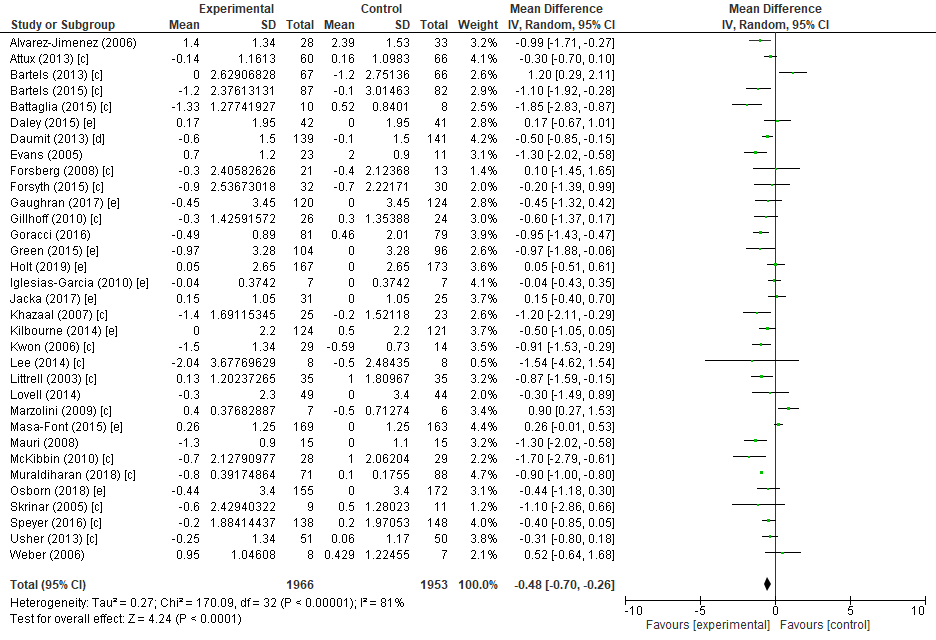


Supplementary Figure 8. Meta-analysis for waist circumference (cms)

**
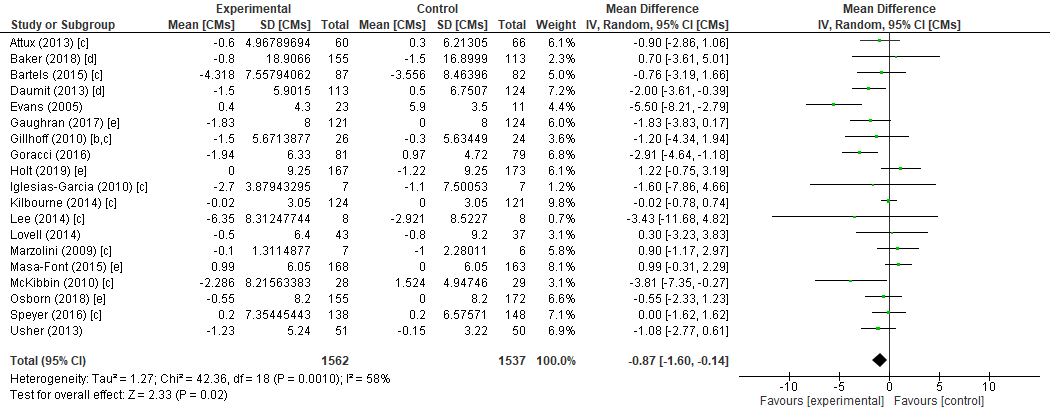
**

Supplementary Table 4. Pooled measures for mental health

| Outcome | Pooled measures |
| --- | --- |
| Depression | Center for Epidemiologic Studies Depression Scale (CES-D)  Beck Depression Inventory (BDI-II)  Calgary Depression Scale (CDSS) Montgomery-Asberg Depression Rating Scale (MADRS)  Patient Health Questionnaire (PHQ-9)  Depression Anxiety and Stress Scale (DASS-D) |
| Anxiety | Beck Anxiety Inventory (BAI)  Generalized Anxiety Disorder (GAD-7)  Depression Anxiety and Stress Scale (DASS-A) |
| Severity of Psychological Symptoms | Brief Psychiatric Rating Scale (BPRS)  Depression Anxiety Stress Scale (DASS) |

Supplementary Figure 9. Meta-analysis for Depression (SMD)


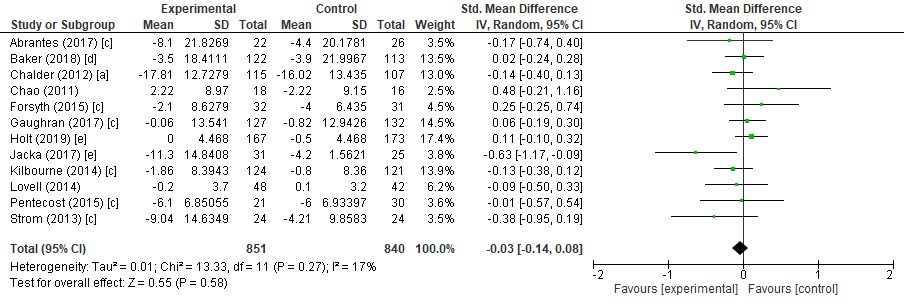


Supplementary Figure 10. Meta-analysis for Anxiety (SMD)


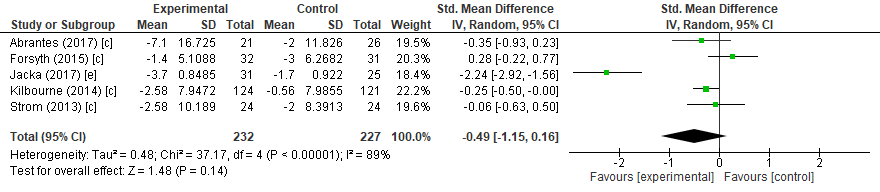


Supplementary Figure 11. Meta-analysis for Severity of Psychological Symptoms (SMD)


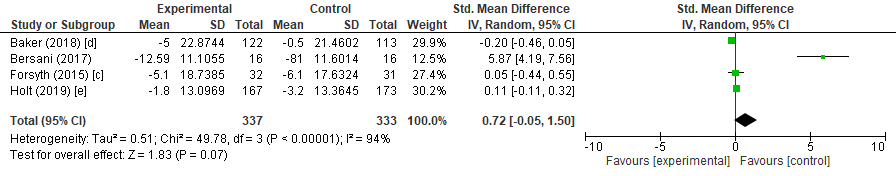

Supplement: Supplementary file 1 — Additional file 1: Supplementary Table 1. Search Strategy (PSYCINFO). Supplementary Table 2. Approaches to calculate required data for meta-analyses when not presented in published studies. Supplementary Table 3. Summary of narrative synthesis. Supplementary Figure 1. Meta-analysis for weight loss (kgs). Supplementary Figure 2. Meta-analysis for weight loss (5% body weight loss). Supplementary Figure 3. Meta-analysis for weight maintenance (kgs). Supplementary Figure 4. Meta-analysis for physical activity (IPAQ - Met Minutes). Supplementary Figure 5. Meta-analysis for diet (fruit serves daily). Supplementary Figure 6. Meta-analysis for diet (vegetable serves daily). Supplementary Figure 7. Meta-analysis for BMI. Supplementary Figure 8. Meta-analysis for waist circumference (cms). Supplementary Table 4. Pooled measures for mental health. Supplementary Figure 9. Meta-analysis for Depression (SMD). Supplementary Figure 10. Meta-analysis for Anxiety (SMD). Supplementary Figure 11. Meta-analysis for Severity of Psychological Symptoms (SMD). [file 13643_2022_2067_MOESM1_ESM.docx]
